# Late Functional Outcomes After Robot-Assisted Radical Prostatectomy: Impact of Baseline and Perioperative Risk Factors

**DOI:** 10.3390/cancers18091406

**Published:** 2026-04-29

**Authors:** Hanka Princlova, Oleg Izmaylov, Minh Nguyet Tranova, Pavel Navratil

**Affiliations:** 1 Department of Urology, University Hospital Hradec Kralove, 50005 Hradec Kralove, Czech Republic; 2 Faculty of Medicine in Hradec Kralove, Charles University, 50005 Hradec Kralove, Czech Republic

**Keywords:** robot-assisted radical prostatectomy, stress urinary incontinence, erectile dysfunction, functional outcomes, prostate cancer

## Abstract

Introduction: Late functional outcomes remain major determinants of quality of life after robot-assisted radical prostatectomy (RARP). Although several baseline and perioperative factors have been linked to postoperative stress urinary incontinence (SUI) and erectile dysfunction (ED), their cumulative effect remains incompletely characterized in large real-world cohorts. Materials and Methods: This retrospective single-center study included 862 consecutive patients undergoing RARP for localized prostate cancer. All endpoints were assessed at a fixed 12-month follow-up visit; therefore, a median follow-up beyond this predefined time point was not applicable. Outcomes were derived from patient-reported information documented during routine follow-up and comprised pad use, ED, and urethral anastomotic stricture. Age, body mass index (BMI), console time, estimated blood loss, and prostate weight were selected a priori based on clinical relevance and uniform availability and were analyzed using univariable and multivariable logistic regression. A simple exploratory composite risk score (0–5 points) was constructed by assigning one point for each predefined adverse factor. Results: At 12 months, 50.0% of patients were pad-free, 85.6% achieved social continence (0–1 pad/day), 14.5% had clinically significant incontinence (>1 pad/day), 71.5% had chart-documented ED, and 1.0% developed urethral anastomotic stricture. In multivariable analysis, age (OR 1.039, 95% CI 1.018–1.059) and prostate weight (OR 1.011, 95% CI 1.004–1.018) independently predicted SUI, while age was the only independent predictor of ED (OR 1.029, 95% CI 1.007–1.050). No predictor of stricture was identified. The composite score showed an exploratory dose–response association with SUI (OR 1.364 per point, 95% CI 1.208–1.541; AUC 0.597) and a weaker association with ED (OR 1.149, 95% CI 1.007–1.313; AUC 0.540). Conclusions: A simple composite score may provide pragmatic exploratory grouping of SUI risk after RARP, but discrimination is modest and interpretation is limited by non-validated outcome assessment and the absence of major confounders, including nerve-sparing status and baseline functional measures.

## Simple Summary

Robot-assisted radical prostatectomy (RARP) is widely used for the treatment of localized prostate cancer, but urinary continence and sexual function after surgery remain highly important to patients. In this retrospective single-center study, we evaluated one-year functional outcomes in 862 consecutive men who underwent RARP. Outcomes were based on patient-reported information recorded during routine follow-up and included pad use, erectile dysfunction, and urethral anastomotic stricture. We analyzed whether commonly available variables such as age, body mass index, console time, blood loss, and prostate weight were associated with these outcomes. Older age and higher prostate weight were linked to worse urinary continence recovery, while age was the only consistent predictor of erectile dysfunction. We also tested a simple exploratory composite risk score, which showed only modest ability to distinguish patients at higher risk, especially for urinary incontinence. These findings suggest that basic perioperative variables may help frame expectations after RARP, but more accurate prediction will require prospective studies including baseline function, nerve-sparing status, and validated patient-reported outcome measures.

## 1. Introduction

Robot-assisted radical prostatectomy (RARP) has evolved from early minimally invasive laparoscopic prostatectomy into the dominant robotic surgical platform for localized prostate cancer in many high-volume centers worldwide. In contemporary practice, the operation is most commonly performed through a multiport transperitoneal approach, although extraperitoneal access may also be used in selected centers. The robotic platform combines stable three-dimensional magnified visualization, articulated wristed instruments, tremor filtration, and precise intracorporeal suturing, thereby facilitating prostate dissection, selective nerve-sparing, and construction of the vesicourethral anastomosis under direct vision [1,2,3,4]. Compared with open and conventional laparoscopic techniques, RARP is associated with reduced blood loss, shorter hospital stay, and improved visualization of pelvic anatomy while maintaining comparable oncological outcomes [1,2,3]. Despite these advantages, long-term functional outcomes remain a major determinant of postoperative quality of life and patient satisfaction.

The broad adoption of RARP has been driven not only by perioperative advantages, but also by the possibility of standardizing fine technical refinements intended to improve postoperative recovery, including graded nerve-sparing, careful bladder neck handling, periurethral tissue preservation, posterior reconstruction, and watertight running anastomosis. At the same time, the relevance of RARP in contemporary urological practice increasingly depends on patient-reported urinary and sexual recovery rather than on perioperative metrics alone, because these outcomes are highly visible to patients and strongly influence satisfaction with treatment [4,5,6,7].

Stress urinary incontinence (SUI) and erectile dysfunction (ED) are the two most frequent functional complications following RARP. Although continence and potency recovery rates have improved over time with refinements in surgical technique, nerve-sparing strategies, and perioperative care, a substantial proportion of patients continue to experience persistent dysfunction at 12 months and beyond [8,9,10]. In addition, urethral anastomotic stricture, although relatively uncommon in the robotic era, represents a clinically relevant complication due to its potential need for repeated interventions and negative impact on urinary function [11].

Previous studies have identified several patient-related and perioperative factors associated with functional recovery after prostatectomy, including age, body mass index (BMI), prostate size, operative time, and blood loss [12,13,14,15]. At the same time, baseline continence and erectile function, nerve-sparing status, comorbidity burden, pathological risk, and intraoperative adverse events are also recognized determinants of recovery and may substantially influence the interpretation of postoperative functional outcomes [4,16,17,18,19]. However, the majority of published data originate from relatively small or heterogeneous cohorts, often focus on single outcomes or isolated predictors, and vary in endpoint definitions. As a result, translating individual risk factors into clinically meaningful preoperative counseling remains challenging.

Importantly, functional outcomes are unlikely to be driven by a single variable alone. Rather, they appear to reflect the cumulative burden of multiple baseline and perioperative risk factors, each contributing incrementally to impaired recovery. While multivariable regression models have been widely used to identify independent predictors, fewer studies have attempted to integrate these factors into a simple and interpretable composite risk framework that could facilitate risk stratification and patient counseling in routine clinical practice [20,21,22]. Such a framework, however, must be interpreted with particular care when key confounders and standardized patient-reported outcome instruments are unavailable.

In large real-world cohorts, such an approach may offer several advantages. First, it allows assessment of dose–response relationships between accumulated risk burden and functional outcomes. Second, it reflects the multifactorial nature of postoperative recovery more accurately than isolated predictors. Finally, a composite score may help identify subgroups of patients at particularly high risk of persistent dysfunction, in whom intensified perioperative strategies, closer follow-up, or early rehabilitation interventions may be warranted.

The aim of the present study was therefore to comprehensively evaluate late functional outcomes after RARP in a large single-center cohort of 862 consecutive patients. Specifically, we assessed the incidence of SUI, ED, and urethral anastomotic stricture at 12 months after surgery and analyzed their association with key patient-related and perioperative variables, including age, BMI, console time, estimated blood loss, and prostate weight. In addition to standard multivariable analyses, we developed a simple composite risk score based on these parameters to examine whether an increasing baseline risk burden is associated with higher rates of postoperative functional complications. By combining detailed outcome assessment with an interpretable risk stratification approach, this study aims to provide clinically relevant data to support patient counseling and risk-adapted postoperative management following RARP.

## 2. Materials and Methods

### 2.1. Study Design and Patient Population

**Figure 1:**
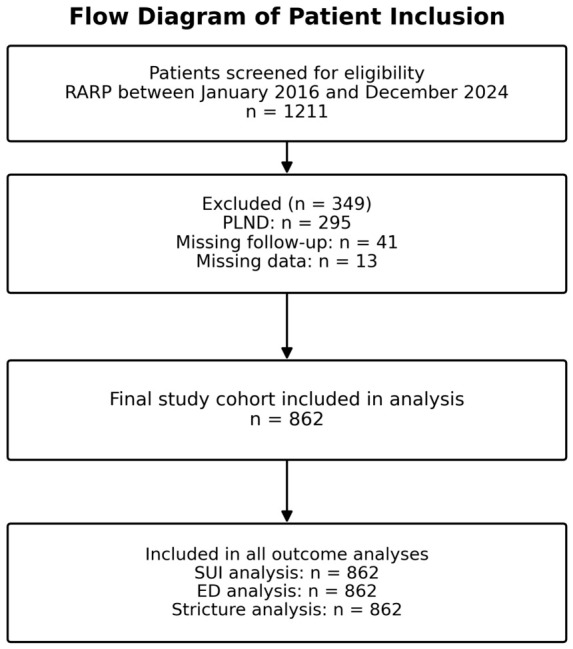
Flow diagram of patient inclusion.

This retrospective observational study included 862 consecutive patients who underwent RARP between January 2016 and December 2024 for clinically localized prostate cancer at a single tertiary referral center. All procedures were performed by experienced surgeons using a standardized robotic approach. Patients undergoing pelvic lymph node dissection or with incomplete baseline/follow-up data were excluded from the respective analyses. Because the study was designed around a predefined fixed 12-month functional assessment, the duration of follow-up for the primary endpoints was uniformly 1 year and a median follow-up beyond this time point was not calculated. The flow diagram in Figure 1 summarizes the overall screening and selection process. Within the final analyzed functional cohort, all 862 patients were alive and evaluable at the 12-month assessment; consequently, 30-day mortality within the analyzed cohort was 0%.

Baseline variables were retrieved from the institutional prostatectomy registry and cross-checked against electronic medical records. Follow-up information was abstracted from scheduled postoperative outpatient visits and, where relevant, endoscopic records documenting the diagnosis and treatment of vesicourethral complications. Because the purpose of this study was to evaluate a clinically usable risk framework, we intentionally focused on parameters that are routinely available in standard RARP datasets and do not depend on specialized imaging post-processing or proprietary questionnaires. Information on nerve-sparing status, validated baseline continence/potency scores, comorbidity burden, and pathological grade was not uniformly available in a format suitable for inclusion in the primary multivariable models.

### 2.2. Surgical Technique and Perioperative Management

All patients underwent RARP using a transperitoneal approach. Nerve-sparing techniques were applied at the discretion of the operating surgeon based on preoperative oncological risk, intraoperative findings, and patient characteristics. Vesicourethral anastomosis was performed using a running suture technique. Perioperative management, including catheterization protocols and postoperative care, followed institutional standards throughout the study period.

Additional technique-level details, including the exact nerve-sparing plane (e.g., incremental, retrograde, or “veil of Aphrodite”), bladder neck preservation, preservation of puboprostatic ligaments or periurethral tissues, deliberate maximal urethral length preservation, and posterior reconstruction (Rocco stitch), were not uniformly documented in the retrospective database and therefore could not be analyzed as covariates in the present study.

All procedures were performed by surgeons experienced in robotic pelvic surgery at a high-volume tertiary referral center. However, surgeon-specific annual volume, chronological case order, and learning-curve effects were not captured as structured variables and were therefore not assessed in the current analysis.

### 2.3. Data Collection and Variables

Clinical, perioperative, and pathological data were prospectively collected and retrospectively analyzed. The following baseline and perioperative variables were included in the analysis:

Age at surgery (years);

BMI (kg/m^2^);

Console time (min);

Estimated blood loss (mL);

Prostate weight (g).

Preoperative membranous urethral length on multiparametric MRI was not routinely measured in a standardized manner during the study period and was therefore unavailable for analysis. Likewise, PI-RADS category and pathological Gleason score/Grade Group were not uniformly available in a structured format suitable for the additional exploratory correlation analyses requested during peer review. The study was predefined as a functional-outcomes analysis rather than a formal pentafecta study; positive surgical margin status, biochemical recurrence data, and standardized perioperative complication grading were therefore not integrated into the primary analytic framework.

### 2.4. Functional Outcome Assessment

Functional outcomes were evaluated at a fixed 12-month follow-up visit after surgery and included SUI, ED, and urethral anastomotic stricture. Endpoints were based on patient-reported information documented during routine postoperative outpatient visits and retrospectively abstracted from the medical record. No validated patient-reported outcome instrument (e.g., IIEF-5 or EPIC-26) was systematically administered within the present retrospective dataset; therefore, the functional endpoints should be interpreted as routine clinical, rather than formal questionnaire-based, outcomes.

The 12-month time point was selected because it is widely used in the literature as a clinically meaningful benchmark for stabilization of early recovery trajectories after radical prostatectomy and is also a common counseling reference for patients and clinicians [5,6,7,23]. Whenever available, pad use was recorded as the average number of pads used per 24 h at the 12-month visit, allowing both binary and graded continence reporting. The distinction between full continence and social continence was considered clinically relevant because many men regard the use of a single safety pad as acceptable recovery, whereas the use of more than one pad per day usually reflects persistent bothersome dysfunction.

### 2.5. Stress Urinary Incontinence (SUI)

SUI was defined as any involuntary urine leakage requiring the use of pads. Continence status was further stratified based on daily pad usage as follows:

Fully continent: 0 pads/day;

Social continence: 1 pad/day;

Clinically significant incontinence: >1 pad/day.

For binary analyses, SUI was defined broadly as the use of ≥1 pad per day. Because this definition conflates the use of a single precautionary pad with more severe leakage, results are also reported separately as pad-free continence (0 pads/day), social continence (0–1 pad/day), and clinically significant incontinence (>1 pad/day).

### 2.6. Erectile Dysfunction (ED)

ED was defined as patient-reported inability to achieve or maintain an erection sufficient for sexual intercourse at 12 months postoperatively. Data on postoperative ED treatment, including phosphodiesterase type 5 inhibitors and intracavernosal injection therapy, were recorded. Because preoperative erectile function and validated questionnaire scores were not uniformly available, this endpoint represents a pragmatic chart-based clinical assessment rather than a formal IIEF-based potency definition.

### 2.7. Urethral Anastomotic Stricture

Anastomotic stricture was defined as a clinically significant narrowing at the vesicourethral anastomosis requiring endoscopic or surgical intervention. Information on stricture treatment and the number of reinterventions was documented.

### 2.8. Composite Risk Score

To assess the cumulative impact of baseline and perioperative risk factors, a simple exploratory composite risk score was developed. One point was assigned for each of the following predefined thresholds:

Age ≥ 70 years;

BMI ≥ 30 kg/m^2^;

Console time ≥ 150 min;

Estimated blood loss ≥ 300 mL;

Prostate weight ≥ 60 g.

The resulting composite score ranged from 0 to 5 points, with higher scores reflecting a greater baseline risk burden. Patients were stratified according to their composite score, and functional outcomes were compared across score categories.

The cutoffs used for the composite score were selected a priori on the basis of common clinical thresholds and the observed distribution of variables in the cohort. The intention was not to generate a mathematically optimized algorithm, but rather to define a pragmatic higher-risk phenotype that could be reproduced in routine practice without additional software. In addition to regression analyses treating the score as an ordinal variable, we also examined outcome frequencies across each score category to assess whether a visible risk gradient was present. Equal weighting was chosen for interpretability, acknowledging that the true contribution of individual variables is likely unequal and that key determinants such as nerve-sparing status, baseline continence, baseline erectile function, comorbidity burden, pathological grade, and intraoperative complications were not represented. Because urethral anastomotic stricture was a rare event, results for this endpoint were interpreted cautiously and considered exploratory.

### 2.9. Statistical Analysis

Continuous variables are presented as the median and interquartile range or mean ± standard deviation, as appropriate. Because several perioperative variables showed skewed distributions and because the analysis focused on between-group differences rather than distributional assumptions of normality, group comparisons for continuous variables were performed using the Mann–Whitney U test. Categorical variables are reported as absolute numbers and percentages; comparisons were performed using the chi-square test when expected cell counts were adequate and Fisher’s exact test when small cell frequencies made exact inference more appropriate.

Univariable and multivariable logistic regression analyses were used to identify predictors of SUI, ED, and urethral anastomotic stricture because all primary endpoints were binary outcomes assessed at a fixed 12-month time point. The five candidate predictors (age, BMI, console time, estimated blood loss, and prostate weight) were prespecified before model construction because they were clinically plausible and uniformly available for the entire cohort; no stepwise or data-driven variable selection procedure was used. Results are reported as odds ratios (ORs) with 95% confidence intervals (CIs), which were included to reflect estimate precision and statistical uncertainty. The association between the composite risk score and functional outcomes was assessed using logistic regression and relative risk estimates stratified by score categories, with patients scoring 0 points serving as the reference group. The composite score analyses were considered exploratory and were intended to test for the presence of a clinically visible risk gradient rather than to validate a prediction tool.

Model discrimination was evaluated using receiver operating characteristic curve analysis and the area under the curve (AUC), as these measures summarize how well the exploratory models distinguish between patients with and without the studied endpoint across decision thresholds. A two-sided *p*-value < 0.05 was considered statistically significant. All statistical analyses were performed using standard statistical software (NCSS version 10; 2015 NCSS LLC).

## 3. Results

### 3.1. Patient Characteristics

A total of 862 patients who underwent RARP were included in the analysis. Baseline demographic and perioperative characteristics are summarized in Table 1. The median age at surgery was approximately in the mid-sixties, with a broad distribution reflecting routine clinical practice. The median BMI was in the overweight range. The median console time and estimated blood loss were consistent with contemporary RARP series, and prostate weight showed substantial interindividual variability.

The mean age was 65.8 ± 7.1 years; the mean BMI was 28.2 ± 3.8 kg/m^2^; the mean console time was 128 ± 40 min; the mean estimated blood loss was 193 ± 178 mL, and the mean prostate weight was 57.3 ± 38.5 g. Taken together, these values indicate that the study population represented a typical contemporary RARP cohort composed predominantly of older, overweight men with moderate perioperative variability and a broad range of prostate sizes.

### 3.2. Functional Outcomes at 12 Months

At 12 months after surgery, 431 patients (50.0%) were fully continent (0 pads/day), 737 (85.6%) achieved social continence (0–1 pad/day), and 125 (14.5%) had clinically significant incontinence (>1 pad/day). When a broad binary definition of SUI was applied, any pad use (≥1 pad/day) was present in 432 patients (50.1%). This binary figure should therefore be interpreted alongside the graded pad-based categories rather than as a direct proxy for bothersome incontinence.

Among patients with persistent urinary incontinence, 8 men (0.9% of the full cohort) ultimately underwent implantation of a male sling or artificial urinary sphincter, reflecting the subgroup with treatment-refractory SUI.

Chart-documented ED at 12 months was reported in 616 patients (71.5%). Among the full cohort, 166 patients (19.3%) received phosphodiesterase type 5 inhibitor therapy and 51 (5.9%) required intracavernosal injection therapy. Because ED assessment was derived from routine clinical documentation rather than a validated instrument and preoperative erectile function was not uniformly available, this proportion should be interpreted as a pragmatic clinical estimate rather than a questionnaire-based potency rate.

Urethral anastomotic stricture was identified in 9 patients (1.0%) and 7/862 patients (0.8%) required surgical revision (Table 2). Owing to the very low event count, all analyses of this endpoint were considered exploratory.

These data indicate that the apparently high binary SUI prevalence largely reflects the inclusion of men using only one safety pad per day; from a pragmatic clinical perspective, the rate of clinically significant incontinence was markedly lower.

### 3.3. Multivariable Logistic Regression Analysis of Predictors of Functional Outcomes

On multivariable logistic regression analysis including age, BMI, console time, estimated blood loss, and prostate weight (Table 3), older age (OR 1.039 per year, 95% CI 1.018–1.059; *p* < 0.001) and larger prostate weight (OR 1.011 per gram, 95% CI 1.004–1.018; *p* = 0.001) were independent predictors of SUI at 12 months. BMI, console time, and blood loss were not independently associated with SUI.

For ED, age remained the only independent predictor in multivariable analysis (Table 4) (OR 1.029 per year, 95% CI 1.007–1.050; *p* = 0.009). Console time showed only a borderline association (OR 1.006 per minute, 95% CI 1.000–1.012; *p* = 0.050), while BMI, blood loss, and prostate weight were not independently associated with erectile function recovery.

No independent predictors of urethral anastomotic stricture were identified in multivariable analysis (Table 5). All estimates were accompanied by wide confidence intervals, consistent with limited statistical power for this rare endpoint.

### 3.4. Composite Risk Score and Functional Outcomes

Patients were stratified according to the composite risk score ranging from 0 to 5 points. The distribution of scores was right-skewed, with the majority of patients scoring 1 or 2 points, and only a small proportion accumulating ≥4 points (Table 6).

The exact distribution of score categories was as follows: 226 patients (26.2%) scored 0, 304 (35.3%) scored 1. 191 (22.2%) scored 2. 101 (11.7%) scored 3. 31 (3.6%) scored 4, and 9 (1.0%) scored 5. Accordingly, 141 patients (16.4%) belonged to the clinically intuitive higher-risk subgroup with a score of at least 3.

A clear exploratory dose–response relationship was observed between the composite risk score and SUI. Compared with score 0, the relative risk of SUI increased across higher score categories and the ordinal model showed a 36% increase in odds per point (OR 1.364, 95% CI 1.208–1.541; *p* < 0.001; Table 7). However, discrimination remained modest (AUC 0.597).

When stratified by composite risk burden (0–5 points), functional outcomes demonstrated a risk-dependent pattern that was most evident for SUI. The gradient for ED was weaker and less consistent, and urethral anastomotic stricture showed no biologically plausible dose-dependent pattern.

For ED, the association with composite risk burden was weaker. Each additional point in the composite score was associated with a 15% increase in odds of ED (OR 1.149, 95% CI 1.007–1.313; *p* = 0.040), but discrimination was limited (AUC 0.540), and category-specific estimates became unstable in the highest score groups because of small patient numbers.

In contrast, no consistent association was observed between the composite risk score and urethral anastomotic stricture.

In category-based analyses, the SUI rate rose from 37.6% in patients with a score of 0 to 50.0%, 52.9%, 67.3%, 58.1%, and 77.8% in patients with scores of 1, 2, 3, 4, and 5, respectively. ED rates increased less consistently, from 67.3% at score 0 to 72.0%, 70.2%, 80.2%, 67.7%, and 100% at scores 1–5. Owing to the very small number of patients with scores 4–5, these highest-category estimates should be interpreted cautiously.

### 3.5. Risk Stratification by Composite Score Categories

When analyzed by individual score categories, SUI was clearly higher from score 3 upward relative to the reference group (score 0). For ED, only score 3 showed a statistically significant difference versus score 0, whereas the remaining categories were either non-significant or trend-level only, consistent with a weaker gradient and limited subgroup size. No statistically significant differences in stricture rates were observed across score categories.

## 4. Discussion

This study examined late functional outcomes after RARP in 862 consecutive patients and explored whether a simple bedside composite of readily available variables could reflect cumulative risk. The principal findings were that (i) age and prostate weight were associated with worse urinary continence recovery, (ii) age was the only consistent predictor of ED in the available models, and (iii) neither the individual variables nor the composite score meaningfully predicted anastomotic stricture. However, these findings must be interpreted cautiously because functional outcomes were derived from routine patient-reported follow-up rather than standardized questionnaires, and the primary models did not include several major confounders such as baseline continence, baseline erectile function, nerve-sparing status, comorbidity burden, or tumor grade.

In addition, the present analysis was intentionally anchored to a fixed 12-month assessment. Therefore, the manuscript addresses one-year functional recovery rather than long-term durability beyond 12 months, and the term “late” should be interpreted in that restricted postoperative context.

The apparently high urinary incontinence burden in this cohort requires contextualization. When any pad use was treated as SUI, 50.1% of men were classified as incontinent at 12 months. Yet this broad definition combines patients using a single precautionary pad with those experiencing clinically relevant leakage. When graded pad use was examined, 50.0% were completely pad-free, 85.6% achieved social continence (0–1 pad/day), and only 14.5% had clinically significant incontinence (>1 pad/day). This distinction is critical because many contemporary series report either 0-pad continence or 0–1-pad social continence rather than any-pad use, and direct comparison without harmonizing definitions can make outcomes appear worse than they actually are [5,23].

Even with this caveat, older age and larger prostate weight were associated with poorer continence recovery, in keeping with prior literature [24,25,26,27,28]. These findings are biologically plausible: age may capture diminished sphincteric reserve and slower neuromuscular recovery, whereas larger glands may be associated with more demanding apical dissection and shorter preserved urethral length. The composite score also showed an observable risk gradient for SUI. Nonetheless, its performance remained modest (AUC about 0.60), which suggests that it should be viewed as a pragmatic descriptive grouping tool rather than a clinically validated prediction model.

At the same time, unmeasured technique-level factors such as the extent and plane of nerve-sparing, bladder neck handling, periurethral tissue preservation, posterior reconstruction, and maximal urethral length preservation may also have materially influenced continence recovery and likely account for part of the unexplained variance in the present models.

Accordingly, we believe the score may still be useful for broad counseling—for example, to distinguish patients with low cumulative perioperative burden from those with several unfavorable features—but not to generate individualized probability estimates. Such patients may merit earlier pelvic floor rehabilitation and more explicit expectation-setting, particularly because structured pelvic floor muscle training can accelerate early continence recovery in selected men [23,29].

The ED results require even greater caution. The observed 71.5% rate is higher than that reported in some contemporary robotic series, but this likely reflects differences in endpoint definition and case mix. In the present study, ED was reconstructed from routine clinical documentation and treatment records rather than from validated instruments such as IIEF-5 or EPIC-26, and baseline erectile function was not uniformly available. In addition, the models did not include nerve-sparing status, which is one of the strongest determinants of postoperative potency. These factors substantially limit comparability with studies that report questionnaire-based potency among men who were potent preoperatively [4,5,6,7,16,17,30,31,32,33].

Within these constraints, age emerged as the only consistent predictor of ED and the composite score showed only a weak association (AUC about 0.54). We therefore do not interpret the ED model as clinically predictive. Rather, it illustrates that basic demographic and perioperative variables alone explain only a small fraction of sexual recovery, which is more strongly shaped by neurovascular preservation, baseline function, metabolic comorbidity, and rehabilitation strategies [4,16,17,30,31,32].

This distinction between urinary and sexual outcomes is important. Continence recovery appears to be influenced, at least in part, by cumulative anatomical and perioperative burden, whereas erectile recovery is more dependent on neurovascular integrity and baseline functional reserve. The weaker and less linear gradient observed for ED in our cohort is concordant with longitudinal quality-of-life studies showing broad heterogeneity in sexual recovery trajectories after prostatectomy [6,7].

The reviewer is also correct that disease aggressiveness and operative complexity deserve attention. Tumor grade and higher-risk disease may constrain nerve-sparing and indirectly worsen functional outcomes through more extensive resection, a concept supported by contemporary RARP series in higher-risk populations [18]. Likewise, intraoperative adverse events or technically difficult surgery may plausibly influence subsequent continence or sexual recovery; recent work has highlighted the importance of systematically reporting intraoperative complications during robotic prostatectomy [19]. In the present retrospective dataset, Gleason score/Grade Group, PI-RADS category, and surgeon-specific experience metrics were not uniformly available in a structured form suitable for additional correlation analyses, and therefore these reviewer-suggested associations could not be tested reliably.

Urethral anastomotic stricture was rare (about 1%) and no meaningful predictor emerged. Given only nine events, this analysis is clearly underpowered and should be considered exploratory. Still, the lack of a consistent association with broad baseline patient characteristics is in line with prior series suggesting that vesicourethral narrowing after RARP may be driven more strongly by local tissue ischemia, anastomotic tension, healing biology, catheter management, and subtle technical factors than by age, BMI, or gland size alone [19,34,35,36,37]. In other words, patient-related risk may be outweighed by procedure-specific and wound-healing mechanisms, which helps explain why published studies have often failed to identify stable patient-level predictors. Clinically, this implies that meticulous anastomotic technique, careful perioperative management, and structured recognition of operative complexity may be more relevant for stricture prevention than simple preoperative risk profiling.

The practical implication of our findings is therefore selective rather than universal. A simplified cumulative score may help frame expectations regarding continence recovery when discussing surgery with patients, but it cannot replace more comprehensive models or individualized clinical judgment. For erectile outcomes in particular, omission of baseline potency, nerve-sparing status, comorbidities, and validated questionnaires makes any simplified score inherently incomplete.

From a practical perspective, the most plausible strategies to reduce complications and improve postoperative quality of life are careful patient selection, technically meticulous surgery, performance of RARP in experienced centers, individualized nerve-sparing when oncologically appropriate, structured pelvic floor rehabilitation, early recognition and treatment of persistent urinary leakage, and proactive sexual rehabilitation including pharmacologic and injection-based therapy when indicated. These measures are supported by the broader RARP literature and are consistent with the view that functional recovery depends on both surgical quality and longitudinal postoperative care.

The simplicity and interpretability of the proposed composite risk score remain strengths. Unlike complex nomograms or web-based longitudinal calculators, it can be calculated immediately from routinely available clinical data. That said, simplicity came at the cost of discrimination, and the equal weighting of arbitrarily chosen cutoffs should be recognized as a deliberate heuristic rather than an optimized statistical solution [33].

Several limitations should be emphasized. The retrospective design introduces potential selection and information bias. Functional endpoints were based on patient-reported information documented in routine care rather than on validated questionnaires, and preoperative urinary and sexual function were not uniformly available. The models lacked nerve-sparing status, comorbidity burden, pathological grade, PI-RADS score, and intraoperative complication data. Detailed technical descriptors such as bladder neck preservation, posterior reconstruction, periurethral tissue preservation, and deliberate urethral length preservation were not captured in a standardized manner, and surgeon learning-curve effects were not analyzed. Preoperative membranous urethral length on multiparametric MRI was unavailable, and the study was not configured for a formal pentafecta analysis because positive surgical margins, biochemical recurrence, and standardized complication grading were not integrated into the predefined functional dataset. In addition, the low incidence of stricture limited power for rare-event inference.

The study also has important strengths. It includes a large consecutive single-center RARP cohort from a high-volume tertiary center, applies a uniform 12-month assessment point, and evaluates urinary continence, sexual function, and stricture within the same analytic framework. This parallel approach allows direct comparison of how the same readily available baseline and perioperative variables behave across different functional domains.

Future studies should combine validated patient-reported outcome instruments, pretreatment functional status, nerve-sparing extent, comorbidity detail, pathological grade, and intraoperative adverse-event reporting to determine whether a more comprehensive—but still clinically usable—model can meaningfully improve prediction after RARP. External validation will be essential before any simplified score can be recommended for routine decision support.

A further practical point is that bedside risk communication after RARP should ideally combine population-level evidence with transparent explanation of uncertainty. In our view, a simplified score may be useful when framed as a conversation aid—helping clinicians explain that continence recovery becomes less favorable as adverse features accumulate—rather than as a deterministic forecast. This distinction is especially important in patients with preserved baseline function and strong interest in nerve-sparing surgery, because individualized recovery may be substantially better or worse than suggested by perioperative variables alone. Prospective studies that integrate standardized PROMs, pathological risk, surgical detail, and rehabilitation data will be necessary to define whether such simplified tools can be safely translated into routine shared decision-making.

## 5. Conclusions

In this large single-center cohort, one-year functional outcomes after RARP showed different patterns of association with baseline and perioperative factors. A simple composite score constructed from routinely available variables demonstrated an exploratory risk gradient for urinary continence, but discrimination was modest and clearly insufficient for stand-alone clinical prediction. Sexual function and urethral stricture were less well explained by the available variables, underscoring the need for future models that incorporate validated baseline function, nerve-sparing status, tumor grade, comorbidity burden, detailed operative technique, imaging-derived parameters such as membranous urethral length, and intraoperative events. The main learning point is that graded, patient-centered reporting of continence and sexual outcomes is more informative than broad binary endpoint definitions alone. The take-home message is that readily available perioperative variables may help frame expectations for continence recovery after RARP, but high-quality counseling and complication reduction still depend on surgical expertise, structured postoperative rehabilitation, and more comprehensive prospective outcome models.

## Figures and Tables

**Table 1:** Baseline Characteristics (n = 862).

Variable	Mean ± SD	Median (IQR)	Range
Age (years)	65.8 ± 7.1	67 (61–71)	41–83
BMI (kg/m^2^)	28.2 ± 3.8	28.0 (25.3–30.5)	18–44
Console time (min)	128 ± 40	125 (110–140)	60–240
Estimated blood loss (mL)	193 ± 178	150 (100–250)	0–2300
Prostate weight (g)	57.3 ± 38.5	50 (41–64)	20–250

**Table 2:** Functional Outcomes 12 Months After RARP.

Outcome	Count	Rate (%)	95% CI
Fully continent (0 pads/day)	431	50	46.7–53.3
Social continence (0–1 pads/day)	737	85.6	83.1–87.8
Clinically significant incontinence (>1 pad/day)	125	14.5	12.2–16.9
Erectile dysfunction	616	71.5	68.4–74.5
Urethral anastomotic stricture	9	1	0.4–1.7

**Table 3:** Multivariable logistic regression for SUI at 12 months.

Variable	Odds Ratio (OR)	95% CI	*p*-Value
Age (per year)	1.039	1.018–1.059	<0.001
BMI (kg/m^2^)	1.026	0.988–1.065	0.185
Console time (per min)	0.999	0.995–1.003	0.622
Estimated blood loss (per mL)	1.001	1.000–1.002	0.145
Prostate weight (per g)	1.011	1.004–1.018	0.001

**Table 4:** Multivariable logistic regression for ED at 12 months.

Variable	Odds Ratio (OR)	95% CI	*p*-Value
Age (per year)	1.029	1.007–1.050	0.009
BMI (per kg/m^2^)	0.966	0.927–1.006	0.092
Console time (per min)	1.006	1.000–1.012	0.05
Blood loss (per mL)	1	0.999–1.001	0.58
Prostate weight (per g)	1.001	0.997–1.005	0.735

**Table 5:** Multivariable logistic regression for urethral anastomotic stricture at 12 months.

Variable	Odds Ratio (OR)	95% CI	*p*-Value
Age (per year)	1.091	0.982–1.212	0.105
BMI (per kg/m^2^)	1.032	0.864–1.234	0.727
Console time (per min)	1.003	0.991–1.016	0.63
Blood loss (per mL)	1	0.996–1.004	0.885
Prostate weight (per g)	0.984	0.951–1.019	0.376

**Table 6:** Composite Risk Score Definition. Each patient received **1 point** for each of the following baseline “risk-raising” factors.

Risk Factor	Threshold	Points
Age	≥70 years	1
BMI	≥30	1
Console time	≥150 min	1
Estimated blood loss	≥300 mL	1
Prostate weight	≥60 g	1

Total possible score: 0–5 points.

**Table 7:** Relative Risk of Postoperative Functional Complications Across Composite Risk Score Levels.

Composite Score	RR–SUI	RR–ED
0	1	1
1	1.33	1.07
2	1.41	1.04
3	1.79	1.19
4	1.54	1.01
5	2.07	1.49

## Data Availability

The data that support the findings of this study are not publicly available due to the sensitive nature of patient data and institutional restrictions. Data may be available from the corresponding author upon reasonable request and with permission of the Institutional Review Board.

## Abbreviations

The following abbreviations are used in this manuscript:

AUC	Area under the curve
BMI	Body mass index
CI	Confidence interval
ED	Erectile dysfunction
RARP	Robot-assisted radical prostatectomy
SUI	Stress urinary incontinence
